# The Employment Quality of Persons with Disabilities: Findings from a National Survey

**DOI:** 10.1007/s10926-023-10113-7

**Published:** 2023-04-12

**Authors:** Faraz Vahid Shahidi, Arif Jetha, Vicki Kristman, Peter M Smith, Monique AM Gignac

**Affiliations:** 1 Institute for Work & Health, 1800-400 University Avenue, Toronto, ON M5G 1S5 Canada; 2 Dalla Lana School of Public Health, University of Toronto, Toronto, ON Canada; 3 EPID@Work Research Institute, Lakehead University, Thunder Bay, ON Canada; 4 Department of Epidemiology and Preventive Medicine, Monash University, Melbourne, Australia

**Keywords:** Disability, Job quality, Employment equity, Inclusion, Precarious employment

## Abstract

**Purpose:**

Labour market integration is a widely accepted strategy for promoting the social and economic inclusion of persons with disabilities. But what kinds of jobs do persons with disabilities obtain following their integration into the labour market? In this study, we use a novel survey of workers to describe and compare the employment quality of persons with and without disabilities in Canada.

**Methods:**

We administered an online, cross-sectional survey to a heterogeneous sample of workers in Canada (n = 2,794). We collected data on sixteen different employment conditions (e.g., temporary contract, job security, flexible work schedule, job lock, skill match, training opportunities, and union membership). We used latent class cluster analysis to construct a novel typology of employment quality describing four distinct ‘types’ of employment: standard, portfolio, instrumental, and precarious. We examined associations between disability status, disability type, and employment quality.

**Results:**

Persons with disabilities reported consistently lower employment quality than their counterparts without disabilities. Persons with disabilities were nearly twice as likely to report low-quality employment in the form of either instrumental (i.e., secure but trapped) or precarious (i.e., insecure and unrewarding) employment. This gap in employment quality was particularly pronounced for those who reported living with both a physical and mental/cognitive condition.

**Conclusion:**

There are widespread inequalities in the employment quality of persons with and without disabilities in Canada. Policies and programs aiming to improve the labour market situation of persons with disabilities should emphasize the importance of high-quality employment as a key facet of social and economic inclusion.

## Introduction

Governments have implemented a wide range of policies and programs with the aim of promoting the social and economic inclusion of persons with disabilities [1, 2]. Among other important tools, labour market integration is a widely accepted strategy for pursuing the goal of inclusion [3, 4]. Whereas employment is a central activity in the lives of most working-age adults – providing a vital source of income, status, structure, meaning, and connection [5–7] – persons with disabilities are much less likely than their counterparts without disabilities to secure and maintain employment [8]. In Canada, for example, only three in five persons with disabilities are actively employed (59%), compared to four in five persons without disabilities (80%) [9]. Similar employment gaps have been observed in other jurisdictions, including the United States and Europe [10, 11].

Previous research on the labour market inclusion of persons with disabilities has focused almost exclusively on overall labour force participation and employment rates, with considerably less attention devoted to understanding what happens following their integration into the labour market [12, 13]. As a result, we know relatively little about the quality of employment obtained by persons with disabilities [14, 15]. The prevailing emphasis on raising overall employment rates overlooks the problem that not all jobs are created equal, and that some jobs offer few of the assumed benefits of paid employment [5, 16, 17]. Simply put, while labour force participation has the potential to promote social and economic well-being, participation alone does not guarantee inclusion – at least not in the form of high-quality employment [18, 19].

Employment quality has deteriorated in Canada and other high-income countries [20]. Over the past several decades, there has been a gradual erosion of the so-called ‘Standard Employment Relationship’ characterized by full-time, permanent, and well-paid employment [20, 21]. Against a backdrop of decline in the relative share of ‘good’ jobs, the prevalence of temporary, low-wage, and other forms of precarious employment has increased steadily with time [22, 23]. Due to the numerous barriers they are known to face in the labour market, persons with disabilities may be particularly vulnerable to the threat of precarious employment [18, 24]. A lack of overall employment opportunities could, for instance, place undue pressure on persons with disabilities to accept any available job – even a low-quality job.

Despite their potential vulnerability to precarious employment, surprisingly little research has been undertaken to evaluate the employment quality of persons with disabilities [14, 15, 25, 26]. Furthermore, existing work in this area has tended to focus on a narrow set of variables – such as hours worked and dollars earned – resulting in a truncated view of employment conditions among persons with disabilities [11]. Employment quality, on the other hand, is an inherently multidimensional construct, meant to capture a broad array of employment conditions including those related to employment stability (e.g., job security), material rewards (e.g., adequate pay), working time arrangements (e.g., a predictable work schedule), career prospects (e.g., opportunities for training and skills development), employment rights (e.g., workplace safety), and power relations (e.g., union representation) [27–29]. There is a need for research that combines these multiple dimensions to provide a more comprehensive picture of employment quality among persons with disabilities.

The goal of this paper is to describe and compare the employment quality of persons with and without disabilities using an explicitly multidimensional approach. Drawing on a novel survey of workers in Canada, we examined how persons with and without disabilities differed across sixteen unique dimensions of employment quality. We also combined the sixteen dimensions into a comprehensive typology of employment quality to assess whether overarching ‘types’ of employment are more or less prevalent among persons with disabilities. We explored these patterns in terms of both disability status (i.e., yes or no) and type of disability (i.e., physical, mental/cognitive, or both).

## Methods

### Study Design and Sample

Data were collected using an online, cross-sectional survey administered in June 2020, as public health restrictions to limit the spread of the first wave of the COVID-19 pandemic were in the process of being relaxed. Participants were recruited from a broadly representative panel of 100,000 Canadians that is maintained by EKOS Research Associates. Panel members were eligible to participate in the study if they were at least 18 years of age, working 12 or more hours per week, and fluent in either English or French. Purposive sampling ensured that at least one third of recruited participants were living with a physical or mental/cognitive condition that created limitations at their job at least some of the time (i.e., a disability). Informed consent was obtained from all participants.

Invitations were sent to 14,570 individuals, of which 51 were determined to have an invalid email. Two reminder emails were sent. A total of 3,842 surveys were returned, yielding a response rate of 26.5%. We excluded 774 respondents who were on temporary leave, on furlough, or working fewer than 12 h per week. We also excluded 227 respondents who were unemployed, retired, or otherwise unattached to the labour market (e.g., due to school or caring responsibilities). Finally, we removed 47 responses missing data on key study variables. This resulted in a final analytic sample of 2,794 study participants.

### Study Measures

*Disability.* Disability status was measured using a modified version of the short disability screening questionnaire (DSQ) designed by Statistics Canada [30]. Three questions asked whether participants “have any [1) physical; 2) mental or cognitive; 3) other] health problems or long-term conditions expected to last six months or more that can make working difficult at least some of the time?” Examples were provided for physical health problems (e.g., seeing, hearing, walking) and mental or cognitive health problems (e.g., learning, concentrating, psychological conditions like anxiety, depression, substance use). Other health problems were manually coded as physical, mental/cognitive, or both physical and mental/cognitive. Based on these questions, we created a variable for disability status (i.e., yes/no) and type of disability (i.e., physical, mental, or both).

*Employment quality.* Employment quality was measured using items adapted from the Employment Precariousness Scale (EPRES) [31], the Employment Precarity Index (EPI) [21], and the Copenhagen Psychosocial Questionnaire (COPSOQ) [32]. Participants were asked about the following employment conditions.

1. Temporary employment: “Do you have a permanent position or a time-limited contract position with your employer?”
2. Part-time employment: “How many hours on average do you work per week?” We defined part-time employment as working < 30 h per week.
3. Job insecurity: “Are you worried about becoming unemployed?” Responses were on a 5-point scale from 1 = Not at all, 2 = To a small extent, 3 = Somewhat 4 = To a large extent and 5 = To a very large extent. We created a binary variable that combined categories 1 to 3 and categories 4 and 5.
4. Income insecurity: “Do your current wages or salary allow you to cover your basic expenses?” Response categories were No/Yes.
5. Long work hours: “How many hours on average do you work per week?” We defined long hours as working 45 h per week or more.
6. Irregular work schedule: “In the past year, which of the following best describes your work schedule?” Responses included “A regular day shift”, “A regular evening shift”, “A regular night shift”, “A rotating shift”, “A split shift”, “On call”, and “An irregular schedule”. Participants were considered to have an irregular work schedule if they reported working a rotating shift, a split shift, on call, or an irregular schedule.
7. Unpredictable work schedule: “Do you know your work schedule at least one week in advance?” Response categories were No/Yes.
8. Flexible work schedule: “To what extent are you free to ask for a change to your hours or work schedule without fear of retaliation or punishment?” Response categories ranged from 1 = “Not at all” to 5 = “To a very large extent”. We created a binary variable that combined categories 1 to 3 and categories 4 and 5.
9. Wage theft: “In the past 12 months, were you always paid in full for the work you did?” Response categories were No/Yes.
10. Gig work: “Would you say that your job is part of the “gig economy”? Response categories were No/Yes.
11. Job lock: “Do you feel ‘locked’ in your current job (i.e., trapped in your job and unable to look for other work)?” Response categories were No/Yes.
12. Skill mismatch: “How much do you believe your current job makes use of your skills and training?” Responses were “My job makes good use of my skills and training”, “I need more training to help me with my job”, and “I don’t get to use my skills and training in this job”. Participants were considered to have skill mismatch if they provided either of the latter two responses.
13. Training opportunities: “Do you have the possibility of learning new things through your work?” Responses were on a 5-point scale ranging from 1 = Not at all to 5 = To a very large extent. We created a binary variable combining categories 1 to 3 and categories 4 and 5.
14. Perceived workplace safety climate: “Would there be negative consequences for you at work if you raised a health and safety concern or raised an employment rights concern with your employer(s)?” Responses were on a 5-point scale ranging from 1 = Not at all to 5 = To a very large extent. We created a binary variable combining categories 1 to 3 and categories 4 and 5.
15. Union membership: “Do you belong to a union or a professional/managerial society at your place of employment that acts as a bargaining unit?” Response categories were No/Yes.
16. Pension benefits. “Does your job or employer provide you with a pension?” Response categories were No/Yes.

We also collected information on age, gender, education, immigration status, and region of residence. These factors were treated as potential confounders of the association between disability and employment quality. Age was collapsed into three groups: 18 to 34 years, 35 to 49 years, and 50 years or older. Gender was measured by asking respondents whether they identify as a man or as a woman. Education was operationalized using three categories: high school or less, some college or university, and college or university degree. Immigrant status was measured by asking respondents whether they were born in Canada. Region of residence was operationalized using four categories: Western Canada (i.e., British Columbia, Alberta, Saskatchewan, and Manitoba), Central Canada (i.e., Ontario and Quebec), Eastern Canada (i.e., New Brunswick, Newfoundland and Labrador, Nova Scotia, and Prince Edward Island), and Northern Canada (i.e., Yukon, Northwest Territories, and Nunavut). Western and Northern Canada were collapsed together due to a small number of participants from Northern Canada.

### Analyses

We calculated descriptive statistics to summarize the distribution of variables. Log binomial regression models examined associations between disability status, disability type, and individual employment conditions. We employed log binomial models instead of logistic models due to the poor interpretability of odds ratios with common outcomes – i.e., outcomes with point prevalence rates > 10%, such as those in the present study [33, 34]. Models were adjusted for age, gender, immigration status, and education. Persons without disabilities were chosen as the reference group. We used latent class analysis (LCA) to construct an empirical typology of overall employment quality, based on the individual dimensions included in the preceding step. LCA can reduce a heterogeneous sample of units into a smaller number of groups that are relatively homogeneous with respect to a given set of characteristics – in this case, employment conditions [35]. We used LCA to place workers into distinct ‘types’ of employment, each characterized by a unique combination of employment conditions [26–28] To determine the most appropriate typology, we estimated multiple LCA models enumerating a progressively larger number of latent groups. To assess the adequacy of model solutions, we used formal fit indices – the Bayesian Information Criterion (BIC), the Akaike Information Criterion (AIC), and the Vuong-Lo-Mendell-Rubin (VLMR) likelihood ratio test – in combination with a substantive interpretation of the resulting outputs [35]. Upon selection of the final typology, descriptive statistics summarized the distribution of individual employment conditions across employment quality types. We also summarized the prevalence of each employment quality type by disability status and disability type. Finally, we used multinomial regression models to examine associations between disability status, disability type, and employment quality. The models were adjusted for age, gender, education, immigration status, and region of residence.

Analyses used Stata 16.0 and Mplus 8.1. Regression estimates are presented in the form of adjusted prevalence ratios (PR) and corresponding 95% confidence intervals (CI).

### Ethics

Ethics Approval for the study was obtained through the University of Toronto Research Ethics Board [REB#39,085].

## Results

Table 1 presents a description of the study sample. Roughly one third (35.2%) of participants reported living with one or more conditions creating a disability at work. Of the 985 participants with disabilities, 415 (42.1%) reported living with a physical disability, 326 (33.1%) reported living with a mental/cognitive disability, and 244 (24.8%) reported living with both a physical and mental/cognitive disability. Compared to participants without disabilities, participants with disabilities were more likely to be women (52.5% versus 45.3%), more likely to be born in Canada (90.2% versus 83.2%), and less likely to have completed a postsecondary degree (70.0% versus 78.3%). Persons who reported a physical disability tended to be older and were more likely to be men, relative to persons who reported either a mental/cognitive disability or both a physical and mental/cognitive disability.

**Table 1:** Descriptive characteristics of the study sample

Characteristic	No Disability		Any Disability		Disability Type				
					Physical		Mental/Cognitive		Both
	n (%)		n (%)		n (%)		n (%)		n (%)
Observations	1,809 (64.8%)		985 (35.2%)		415 (14.8%)		326 (11.7%)		244 (8.7%)
Age									
18 to 34	642 (35.5%)		320 (32.5%)		80 (19.3%)		151 (46.3%)		89 (37.5%)
35 to 49	615 (34.0%)		355 (36.0%)		130 (31.3%)		130 (39.9%)		95 (38.9%)
50 or above	552 (30.5%)		310 (31.5%)		205 (49.4%)		45 (13.8%)		60 (24.6%)
Gender									
Men	990 (54.7%)		468 (47.5%)		218 (52.5%)		148 (45.4%)		102 (41.8%)
Women	819 (45.3%)		517 (52.5%)		197 (47.5%)		178 (54.6%)		142 (58.2%)
Education									
Postsecondary degree	1,417 (78.3%)		690 (70.0%)		286 (68.92%)		242 (74.2%)		162 (66.4%)
Some postsecondary	266 (14.7%)		193 (19.6%)		77 (18.6%)		63 (19.3%)		53 (21.7%)
Secondary degree or less	126 (7.0%)		102 (10.4%)		52 (12.5%)		21 (6.4%)		29 (11.9%)
Immigration Status									
Born in Canada	1,505 (83.2%)		889 (90.2%)		377 (90.8%)		293 (89.9%)		219 (89.8%)
Not born in Canada	304 (16.8%)		96 (9.8%)		38 (9.2%)		33 (10.1%)		25 (10.2%)
Region									
Western and Northern Canada	538 (29.7%)		327 (33.2%)		144 (34.7%)		98 (30.1%)		85 (34.8%)
Central Canada	1,119 (61.9%)		551 (55.9%)		224 (54.0%)		198 (60.7%)		129 (52.9%)
Eastern Canada	152 (8.4%)		107 (10.9%)		47 (11.3%)		30 (9.2%)		30 (12.3%)

Table 2 shows the weighted prevalence of employment conditions across study groups, as well as fully adjusted negative binomial regression models estimating associations between study variables. Persons with disabilities reported lower quality employment conditions across a large majority of dimensions, including significantly higher rates of temporary employment, part-time employment, job insecurity, income insecurity, gig work, wage theft, job lock, and skill mismatch. They were also significantly less likely to report flexible work schedules, training opportunities, and positive workplace safety climates. Conversely, persons with disabilities were more likely to report membership in a union or professional association. We observed no differences between persons with and without disabilities in the prevalence of long working hours, irregular or unpredictable work schedules, and pension benefits. These associations generally held across different types of disability. However, persons living with both a physical and mental/cognitive disability tended to report greater levels of employment adversity than persons living with only one or the other type of disability.

**Table 2:** Prevalence and adjusted prevalence ratios of employment conditions, by disability status and disability type

	Temporary Employment		Part-Time Employment		Job Insecurity		Income Insecurity	
	%	PR (95% CI)	%	PR (95% CI)	%	PR (95% CI)	%	PR (95% CI)
Disability Status								
No disability	9.1%	Reference	8.4%	Reference	11.9%	Reference	5.6%	Reference
Any disability	11.1%	1.26 (1.00-1.59)	11.7%	1.27 (1.01–1.60)	17.7%	1.52 (1.27–1.83)	12.2%	2.06 (1.60–2.66)
Disability Type								
Physical	10.6%	1.30 (0.93–1.80)	11.1%	1.15 (0.84–1.58)	14.5%	1.34 (1.02–1.75)	11.9%	1.97 (1.42–2.74)
Mental/Cognitive	10.2%	1.08 (0.76–1.55)	10.1%	1.19 (0.83–1.70)	18.5%	1.52 (1.17–1.97)	8.6%	1.52 (1.01–2.27)
Both	13.2%	1.46 (1.01–2.01)	14.8%	1.58 (1.13–2.21)	22.3%	1.80 (1.39–2.35)	17.7%	2.91 (2.09–4.07)
	**Long Hours**		**Irregular Schedule**		**Unpredictable Schedule**		**Flexible Schedule**	
	%	PR (95% CI)	%	PR (95% CI)	%	PR (95% CI)	%	PR (95% CI)
Disability Status								
No disability	19.1%	Reference	16.5%	Reference	9.4%	Reference	49.2%	Reference
Any disability	20.4%	1.10 (0.94–1.28)	18.9%	1.12 (0.95–1.33)	10.7%	1.18 (0.93–1.48)	39.9%	0.82 (0.74–0.90)
Disability Type								
Physical	20.5%	1.05 (0.85–1.29)	18.0%	1.04 (0.82–1.31)	9.7%	1.10 (0.79–1.54)	40.1%	0.81 (0.71–0.93)
Mental/Cognitive	19.3%	1.09 (0.86–1.38)	17.1%	1.07 (0.82–1.39)	11.1%	1.18 (0.84–1.66)	43.9%	0.90 (0.78–1.04)
Both	21.7%	1.21 (0.93–1.56)	22.7%	1.34 (1.04–1.74)	12.1%	1.28 (0.89–1.85)	34.1%	0.70 (0.58–0.85)
	**Gig Work**		**Wage Theft**		**Job Lock**		**Skill Mismatch**	
	%	PR (95% CI)	%	PR (95% CI)	%	PR (95% CI)	%	PR (95% CI)
Disability Status								
No disability	6.3%	Reference	6.5%	Reference	27.2%	Reference	18.0%	Reference
Any disability	9.8%	1.53 (1.18-2.00)	10.7%	1.66 (1.29–2.14)	39.3%	1.44 (1.29–1.61)	28.5%	1.60 (1.39–1.84)
Disability Type								
Physical	8.4%	1.32 (0.91–1.91)	9.0%	1.47 (1.03–2.10)	35.0%	1.33 (1.14–1.55)	21.8%	1.31 (1.06–1.62)
Mental/Cognitive	6.4%	1.04 (0.66–1.65)	7.8%	1.15 (0.75–1.74)	37.4%	1.35 (1.14–1.58)	30.6%	1.63 (1.34–1.97)
Both	16.5%	2.54 (1.82–3.56)	17.6%	2.71 (1.96–3.77)	49.3%	1.77 (1.52–2.07)	37.2%	2.00 (1.64–2.43)
	**Training Opportunities**		**Positive Safety Climate**		**Union Membership**		**Pension Benefits**	
	%	PR (95% CI)	%	PR (95% CI)	%	PR (95% CI)	%	PR (95% CI)
Disability Status								
No disability	57.2%	Reference	79.2%	Reference	34.2%	Reference	59.5%	Reference
Any disability	48.6%	0.88 (0.82–0.95)	64.6%	0.81 (0.77–0.86)	40.4%	1.17 (1.06–1.29)	57.9%	0.98 (0.92–1.04)
Disability Type								
Physical	45.4%	0.83 (0.75–0.93)	71.7%	0.89 (0.83–0.95)	44.3%	1.29 (1.13–1.45)	58.2%	0.98 (0.90–1.07)
Mental/Cognitive	56.4%	0.98 (0.89–1.09)	66.2%	0.84 (0.77–0.91)	35.6%	1.04 (0.89–1.22)	57.8%	0.97 (0.88–1.07)
Both	43.6%	0.80 (0.69–0.92)	50.2%	0.64 (0.56–0.73)	40.0%	1.15 (0.98–1.36)	57.6%	0.97 (0.87–1.09)

Notes: PR = Prevalence Ratio; CI = Confidence Interval; Estimates are adjusted for age, gender, education, immigration status, and region of residence.

LCA models enumerating a progressively larger number of latent groups suggested that the optimal solution lay between three and six classes. Model solutions with seven or more classes extracted groups that were negligible in size (i.e., less than 2% of the sample). These solutions were therefore not considered. The VLMR likelihood ratio tests were consistently significant (p < 0.01) across sequential models, suggesting that model fit improved as the number of enumerated classes increased. However, AIC and BIC values plateaued beyond the four-class solution, indicating little improvement in in statistical fit beyond this solution. We therefore selected the four-class solution as our typology of choice.

Table 3 shows the weighted prevalence of individual employment conditions across employment quality types. We labeled the four types: standard employment, portfolio employment, instrumental employment, and precarious employment [28, 29]. Standard employment was the largest group, representing 38% of the sample. It was characterized by generally favourable employment conditions. Workers described employment providing high levels of job and income security as well as regular and predictable work schedules. They also reported high degrees of flexibility, opportunities for training, and positive environments in which to raise concerns about their health, safety, and rights as workers. A majority of this group belonged to a union or professional association and all respondents reported having an employer-sponsored pension benefit. Standard employment could be summarized as ‘secure and rewarding’.

**Table 3:** Prevalence of employment conditions, by employment quality type

Employment Conditions	Employment Quality Type			
	Standard Employment	Portfolio Employment	Instrumental Employment	Precarious Employment
	n = 1,072 (38.0%)	n = 732 (26.5%)	n = 446 (15.5%)	n = 544 (20.0%)
Temporary Employment	7.2%	11.8%	3.2%	19.2%
Part-Time Employment	5.4%	4.0%	12.7%	22.0%
Job Insecurity	3.2%	3.3%	5.6%	55.6%
Income Insecurity	1.0%	4.6%	4.8%	29.3%
Long hours	19.6%	23.7%	18.6%	15.3%
Irregular Schedule	12.9%	18.1%	16.8%	26.3%
Unpredictable Schedule	4.2%	11.5%	7.6%	20.2%
Flexible Schedule	58.4%	61.3%	10.4%	29.5%
Gig Work	2.3%	9.0%	1.7%	22.8%
Wage Theft	4.5%	5.8%	8.8%	18.2%
Job Lock	11.4%	11.6%	74.0%	62.0%
Skill Mismatch	6.5%	9.6%	45.9%	47.5%
Training Opportunities	76.4%	65.1%	18.7%	24.7%
Positive Safety Climate	92.6%	87.8%	48.3%	38.2%
Union Membership	52.6%	8.6%	69.4%	15.0%
Pension Benefits	100.0%	1.0%	94.5%	25.5%

Portfolio employment was the second largest group, covering 26.5% of the sample. It was also characterized by largely favourable employment conditions, including high rates of job security, income security, flexibility, and opportunities for training. However, relative to those in standard employment, workers in this group had a higher probability of temporary employment and were more likely to report long, irregular, or unpredictable work schedules. Compared to other groups, respondents reported the lowest rates of union or association membership and employer-sponsored pension benefits. Portfolio employment could be summarized as ‘secure but demanding’.

The third group, comprising 15.5% of the sample, was labeled instrumental employment. It described employment situations affording high levels of job and income security as well as regular and predictable work schedules, but little else in the way of opportunities and rewards. A vast majority of workers in this group reported being employed on a permanent basis. They also reported the highest rates of membership in a union or professional association. However, compared to other groups, workers were more likely to feel trapped in their job and least likely to perceive opportunities for training and growth. They also had a high probability of perceiving mismatch between the skills they have and those that are required for the job. Instrumental employment could be summarized as ‘secure but trapped’.

The fourth and final group, accounting for 20% of the sample, was labeled precarious employment. It comprised workers who reported consistently unfavourable employment conditions across all sixteen indicators. Workers reported the highest rates of temporary employment, part-time employment, irregular schedules, unpredictable schedules, gig work, and skill mismatch. They were more likely than other groups to report job insecurity, income insecurity, and wage theft. A small minority of these workers belonged to union or professional association, and most did not consider their workplace to be an environment where they could raise concerns about their health, safety, and rights as workers. Precarious employment could be summarized as ‘insecure and unrewarding’.

Table 4 shows the weighted prevalence of employment quality types across study groups. Compared to persons without disabilities, persons with disabilities were less likely to fall in the Standard Employment and Portfolio Employment groups, and more likely to fall in the Instrumental Employment and Precarious Employment groups. This was especially true for persons reporting both a physical and mental/cognitive disability.

**Table 4:** Prevalence of employment quality types, by disability status

	No Disability	Any Disability	Disability Type		
			Physical	Mental/Cognitive	Both
**Employment Quality Type**	%	%	%	%	%
Standard Employment	42.5%	29.7%	32.5%	31.3%	22.5%
Portfolio Employment	28.9%	22.3%	23.4%	24.6%	17.1%
Instrumental Employment	13.0%	20.0%	19.4%	17.9%	24.2%
Precarious Employment	15.6%	28.0%	24.7%	26.2%	36.2%

Table 5 presents results from fully adjusted multinomial regression models estimating associations between disability status, disability type, and the employment quality typology. Standard employment was selected as the base outcome category. The probability of portfolio (versus standard) employment was similar between persons with and without disabilities (PR: 1.09, CI: 0.88–1.35). This finding held across all three disability types. By contrast, the probability of instrumental (versus standard) employment was twice as high among persons with disabilities (PR: 2.14, CI: 1.69–2.70). Similarly, the probability of precarious (versus standard) employment was two and a half times higher among persons with disabilities (PR: 2.49, CI: 1.99–3.10). Consistent with our earlier findings, these associations were strongest among persons who reported living with both physical and mental/cognitive disabilities. Thus, in the latter group, the probability of instrumental (versus standard) employment was more than three times higher (PR: 3.35, CI: 2.25–4.99) and the probability of precarious (versus standard) employment was more than four times higher (PR: 4.36, CI: 3.01–6.33) relative to their counterparts without disabilities.

**Table 5:** Associations between disability status, disability type, and employment quality

	Portfolio versus Standard	Instrumental versus Standard	Precarious versus Standard
	PR (95% CI)	PR (95% CI)	PR (95% CI)
Disability Status			
No disability	Reference	Reference	Reference
Any disability	1.09 (0.88–1.35)	2.14 (1.69–2.70)	2.49 (1.99–3.10)
Disability Type			
Physical	1.02 (0.76–1.36)	1.93 (1.41–2.63)	1.97 (1.45–2.67)
Mental/Cognitive	1.17 (0.85–1.61)	1.80 (1.26–2.56)	2.18 (1.57–3.02)
Both	1.12 (0.73–1.71)	3.35 (2.25–4.99)	4.36 (3.01–6.33)

Notes: PR = Prevalence Ratio; CI = Confidence Interval; Estimates are adjusted for age, gender, education, immigration status, and region of residence.

## Discussion

Previous research has noted a large and persisting employment gap between persons with and without disabilities [9–11]. Starting from the premise that not all jobs are created equal – and that having any job is not the same as having a good quality job – our study moves this literature forward by examining whether analogous gaps exist in the quality of employment obtained by persons with disabilities following their integration into the labour market. We are among the first to address this question using an explicitly multidimensional approach. By taking this approach, we have provided the most comprehensive picture yet of the employment quality of persons with disabilities in Canada. Our findings point to the presence of widespread inequalities in the employment quality of Canadians with and without disabilities, suggesting that there is still a long way to go for ‘full participation’ in the labour market. These findings have implications for research, workplace practices, and policy in the areas of disability and employment.

Consistent with our multidimensional approach, we observed inequalities not only with respect to conventional indicators of employment quality, such as hours worked and dollars earned, but across an extensive set of employment conditions ranging from job security and flexible work arrangements to skill match and opportunities for training. Taking all sixteen of our dimensions into account, persons with disabilities were nearly twice as likely as their non-disabled counterparts to report low-quality employment in the form of either instrumental (i.e., secure but trapped) or precarious (i.e., insecure and unrewarding) employment. Notably, we observed the largest gaps among those who reported living with both a physical and mental/cognitive disability. These findings highlight the importance of adopting a broad and comprehensive definition of labour market inclusion, and reinforce the need to better distinguish between employment *status* (i.e., having any job) and employment *quality* (i.e., having a good quality job) as related but still distinct aspects of inclusion [18, 19, 36].

Previous studies have documented how persons with disabilities experience barriers not only with respect to obtaining a job, but also with respect to the terms and conditions of their employment [11]. Literature from comparable jurisdictions, such as the United States and Australia, suggests that persons with disabilities experience consistently lower quality employment than their counterparts without disabilities, including fewer hours, lower pay, less job security, and fewer benefits [14, 15, 25, 26, 37–39]. Whereas previous studies focused on one or two dimensions of employment, resulting in a truncated view of the problem, our analysis incorporated a wide range of variables to present a more complete picture of the employment conditions experienced by persons with disabilities. A key advantage of this approach is that we are better able to identify not only where gaps exist between persons with and without disabilities, but also where along the spectrum of employment conditions these gaps are largest and smallest. For instance, we observed larger gaps in subjective employment conditions (e.g., job security, income security, job lock, and skill match) than objective employment conditions (e.g., temporary employment, part-time employment, and regular work schedule). Further research is needed to explain this divergence across the subjective and objective dimensions of employment, as well as its implications for the measurement of employment quality among workers in general and workers with disabilities in particular [40–42]. By incorporating a diverse set of employment variables, our analysis can also inform strategies to improve the working lives of persons with disabilities. Beyond encouraging greater attachment to the labour market, our findings emphasize the importance of job security, wages, flexible work arrangements, job fit, and training opportunities as particularly meaningful intervention targets in need of improvement. Addressing these gaps will require a combination of societal (e.g., employment standards legislation) and organizational (e.g., inclusive workplace practices) interventions.

Using a typological approach, our study also serves to demonstrate how multiple interacting dimensions of work produce distinct ‘types’ of employment that we labeled standard, portfolio, instrumental, and precarious employment. The typology helped us to organize a complex array of factors and highlighted that the various employment ‘types’ are unevenly distributed across labour market groups, including persons with and without disabilities [28, 29, 43, 44]. It was not just that persons with disabilities experienced disadvantage with respect to one or another dimension of employment, but they were also more likely to experience negative *overall* employment arrangements, including seemingly dead-end jobs providing little hope of career advancement and precarious jobs that offered minimal security, stability, and pay. In fact, our findings indicate that inequalities in overall employment quality were substantially larger than those observed along individual dimensions of work, suggesting that previous literature in this area may have underestimated both the nature and the extent of the employment gap between persons with and without disabilities. Future research should test the ecological validity of the typology and further strive to capture the full breadth and depth of the employment experiences of persons with disabilities by drawing on and even extending the multidimensional framework adopted here. Follow up studies should also be conducted to determine whether our findings are generalizable to other high-income jurisdictions (e.g., the United States, Europe, and Australia) and whether they hold in low- and middle-income countries, which have markedly different legislative and labour market contexts with important implications for the employment of persons with disabilities.

Our study did not gather information on employment preferences. Consequently, we had no means of ascertaining whether persons with disabilities prefer certain employment conditions over others. Nevertheless, our findings should be interpreted in light of a growing body of evidence indicating that persons with disabilities desire high-quality employment and share the same perspectives as persons without disabilities on the value and importance of key employment characteristics, such as job and income security [44]. Notwithstanding a lack of information on worker preferences, the results of the present study point to a pattern of responses indicating that many persons with disabilities experience employment conditions that are not of their choosing. For example, it is hard to imagine a ‘preference’ for job insecurity, income insecurity, wage theft, job lock, skill mismatch and a lack of training opportunities – all of which were more commonly reported by persons with disabilities. Researchers have noted that persons with disabilities might actively select into certain forms of non-standard employment (e.g., part-time, temporary, and gig work) given that fewer hours and more flexible contracts could be of special benefit to individuals balancing the obligations of employment with personal health needs [45, 46]. While persons with disabilities might prefer non-standard employment arrangements because they afford greater flexibility, it is worth nothing that such arrangements cluster together with a number of indisputably adverse employment conditions. Thus, in our typology, the group of workers that reported the highest rates of temporary, part-time, and gig work (i.e., the ‘precarious employment’ group) also reported the greatest levels of job insecurity, income insecurity, and unpredictability. This finding suggests that meeting and accommodating the employment needs of persons with disabilities will require striking a better balance between flexibility and stability – to ensure that the former does not come at the expense of the latter [45]. Future research should explore these issues by incorporating measures that explicitly assess worker preferences [44]. Preferences aside, it could very well be that some study participants obtained poor quality jobs before experiencing their disability, and that the precarious nature of their jobs in turn led to a disabling injury or illness [47]. Due to the cross-sectional study design, however, we were unable to explore this complexity. Longitudinal research could be beneficial in this regard, allowing a view of change over time in both disability status and employment quality.

Strengths of the present study include the following. First, our observations are drawn from a heterogeneous and broadly representative panel of Canadian workers. Second, through purposive sampling methods, we recruited persons with diverse experiences of disability, enabling a disaggregated approach accounting for both disability status and type of disability. Finally, compared to previous work in this area, we adopted a more comprehensive and multidimensional approach to the measurement of employment quality. At the same time, our findings and the conclusions we have drawn should be situated in the context of some key limitations. Despite the broadly representative panel from which the study participants were drawn, our survey only returned a response rate of 26.5%. This response rate is comparable to other surveys of this kind. Nevertheless, it introduces the potential for selection bias in our analytic sample. For instance, it is probable that certain groups of workers, such as those living with severe disabilities, were less likely than others to participate in the panel or respond to our invitation, leading to further problems of generalizability. These limitations of the sample should be weighed against the fact that population-based employment surveys in Canada – such as the flagship Labour Force Survey and recent Survey on Quality of Employment – lack measures of disability status and were therefore not suitable for our purposes. Second, as noted above, the cross-sectional nature of the data prohibited us from determining whether participants experienced their disability before or after entering their most recent employment situation. As a result, we were unable to test the directionality of observed associations between disability and employment quality. Finally, data were collected a few months after the onset of the global COVID-19 pandemic, raising concerns that our findings may reflect employment and other surrounding conditions specific to this exceptional period. Indeed, for many of the same reasons discussed in the text above, persons with disabilities were particularly vulnerable to the labour market impacts of the COVID-19 pandemic and associated policy responses [48]. It is worth noting, however, that our survey was administered during a period in which public health restrictions were being lifted and a large majority of Canadians were attending their usual workplaces [49]. At the same time, the growing availability of telework arrangements in the aftermath of the pandemic raises important questions about the labour market integration and workplace accommodation of persons with disabilities [50]. It will be important for future research to examine how the increasing prevalence of home-based work is affecting the labour market and employment outcomes of persons with disabilities.

## Conclusion

The present study demonstrates that persons with disabilities experience inequalities not only with respect to obtaining paid employment, but also with respect to the quality of employment opportunities available to them following their integration into the labour market. Given the declining share of ‘good’ jobs and the increasing prevalence of precarious employment, a narrow focus on employment participation (as an end in itself) has the potential to exacerbate rather than mitigate the problem of social and economic exclusion. Policies and programs aiming to improve the labour market situation of persons with disabilities should emphasize the importance of high-quality employment as a key ingredient of inclusion.

## Data Availability

The datasets generated during and/or analysed during the current study are available from the corresponding author on reasonable request.
